# Mucosal Hub Bacteria as Potential Targets for Improving High-Fat Diet-Related Intestinal Barrier Injury

**DOI:** 10.1155/cjid/3652740

**Published:** 2024-11-27

**Authors:** Li Shao, Binbin Zhang, Yu Song, Zhe Lyu, Weishi Zhang, Wenjun Yang, Jinlong Fu, Jie Li, Junping Shi

**Affiliations:** ^1^School of Clinical Medicine, Hangzhou Normal University, The Affiliated Hospital of Hangzhou Normal University, Hangzhou 311121, Zhejiang, China; ^2^Institute of Hepatology and Metabolic Diseases, Hangzhou Normal University, Hangzhou 310015, Zhejiang, China; ^3^Institute of Translational Medicine, The Affiliated Hospital of Hangzhou Normal University, Hangzhou 311121, Zhejiang, China; ^4^Department of Hepatology, 2nd Affiliated Hospital of Zhejiang Chinese Medical University, Hangzhou 310053, Zhejiang, China; ^5^Department of Stomatology, The First Affiliated Hospital, College of Medicine, Zhejiang University, Hangzhou 310003, Zhejiang, China; ^6^Department of Otolaryngology, Affiliated Hospital 2 of Nantong University, Nantong 226001, Jiangsu, China; ^7^Department of Gastroenterology and Hepatology, The Affiliated Hospital of Hangzhou Normal University, Hangzhou 310015, Zhejiang, China; ^8^Department of Infectious Diseases, Nanjing Drum Tower Hospital, The Affiliated Hospital of Nanjing University Medical School, Nanjing, Jiangsu, China

**Keywords:** high-fat diet, hub bacteria, intestinal barrier injury, mucosal microbiome

## Abstract

**Background:** Intestinal barrier injury contributes to multiple diseases such as obesity and diabetes, whereas no treatment options are available.

**Methods:** Due to close interactions between mucosal microbiome and intestinal barrier, we evaluated the potential of mucosal bacteria in providing targets for high-fat diet (HFD)–related intestinal barrier injury. Whole-genome metagenomics was used to evaluate mucosal microbiome, while intestinal barrier injury was estimated using serum LPS, FITC-dextran intensity, and ZO-1 protein.

**Results:** We found that HFD induced significant fat accumulation in epididymal tissue at weeks 4 and 12, while ALT, LDL, and TC increased at week 12. Intestinal barrier injury was confirmed by elevated serum LPS at both weeks, upregulated FITC-dextran intensity, and decreased ZO-1 protein at week 12. Fourteen species such as *Phocaeicola vulgatus* differed in HFD-fed mice. The co-occurrence network of mucosal microbiome shifted from scale-free graph in controls to nearly random graph in HFD-fed mice. Besides, 10 hub bacteria especially *Bacteroides ovatus* decreased drastically in both mucosal and fecal samples of HFD-fed mice, correlated with intestinal permeability, ALT, and KEGG pathways such as “Mitochondrial biogenesis” and “metabolism”. Moreover, *Bacteroides ovatus* has been confirmed to improve intestinal barrier function in a recent study.

**Conclusion:** Mucosal hub bacteria can provide potential targets for improving HFD-related intestinal barrier function.

## 1. Introduction

Intestinal barrier injury has been found to be an important etiology of diseases such as nonalcoholic fatty liver disease (NAFLD), obesity, and diabetes [1–3]. In this case, the rapidly multiplying pathogenic bacteria, bacterial metabolites, and extracellular vesicles resulting from dysregulated gut microbiota will enter the portal vein via the impaired intestinal barrier, which will then activate a cascade of immune responses, cause organ inflammation, and ultimately promote disease progression [1, 4]. Nowadays, microbial DNA enrichment has been found to promote liver steatosis and fibrosis in NASH [4], to cause myocardial inflammation and impair cardiac contractility [5], as well as to promote islet *β* cell abnormalities, adrenomedullary inflammation, and hypertension in obese mice [6, 7]. Therefore, enhancing intestinal barrier function may inhibit the progression and complications of related diseases from the source [8]. However, no treatment options are available targeting on intestinal barrier injury.

Nowadays, several probiotics such as *Akkermansia muciniphila*, *Lactobacillus plantarum MA2*, and *Lactobacillus plantarum NA 136* have shown efficacy in improving mucosal barrier function by regulating intestinal microbiome [9–11]. Current probiotics are mainly from fecal microbiota differential between diseased and nondiseased populations. However, differential microbiota are highly variable in diseased states [12], resulting in inconsistent performance of current probiotics in clinical trials with different populations [13]. New probiotics for intestinal barrier regulation that are more robust and global in terms of microbial community regulation are required.

Compared to the surface knowledge provided by differential bacteria, inferring deeper relationships between the microorganisms of the microbial community based on network theory may provide additional insights into the forces that shape the ecosystem, especially the hub bacteria that are crucial in maintaining the structure and robustness of networks [14]. Till now, network analysis has been proved valuable in providing rectum keystone microbiota in modulating the microbial community and growth performance in goat model [15], and in identifying hub bacteria *Lachnospiracease UCG-001* as the potential microbial target for treating Alzheimer's disease [16]. On the other hand, mucosal bacteria interact with, and affect the intestinal barrier in a much more direct way, since they colonize the mucus layer, and form a “biological wall” by attaching themselves to the surface of the intestinal epithelial cells. It is proposed that microbial interaction with intestinal epithelial cells at the epithelial barrier defines the immune responses in the region, and implicates in the maintenance of epithelial barrier function [17]. The regional changes in intestinal permeability in cirrhosis have also been confirmed to be associated with mucosal bacteria [18]. Such results indicate that mucosal hub bacteria in microbial networks may provide invaluable targets for intestinal barrier regulation.

Unlike the bumper harvest in fecal microbiome, few research progresses have been made in the association between mucosal hub bacteria and intestinal barrier injury. Therefore, we tried to evaluate the detailed reshape of mucosal microbiome induced by HFD, as well as the associations between mucosal hub bacteria and HFD-related intestinal barrier injury. The aim of the study is to evaluate the potential of mucosal hub bacteria in providing targets for improving intestinal barrier function based on whole-genome metagenomics.

## 2. Materials and Methods

### 2.1. Animal Care and Experimental Design

**Figure 1:**
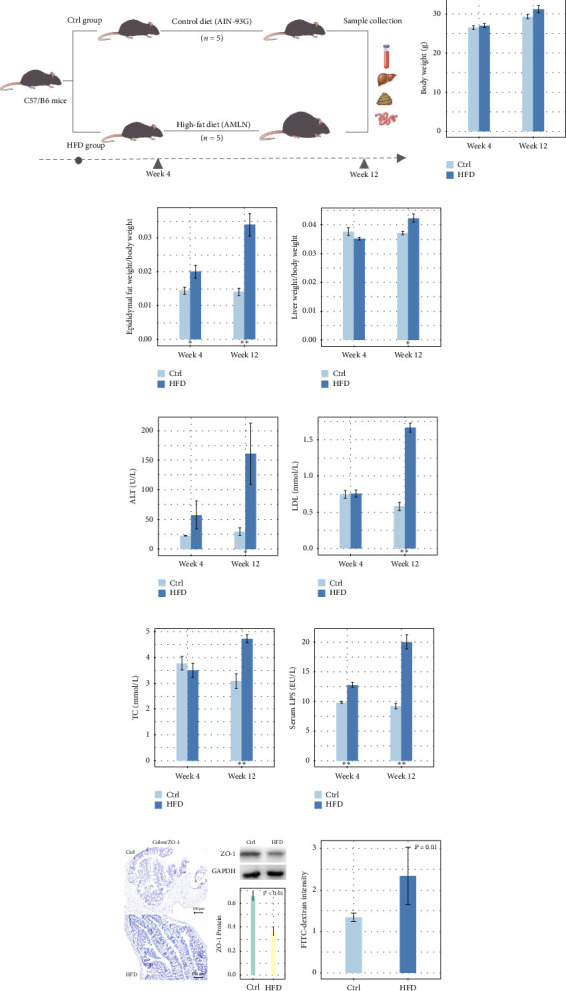
(a) A schematic diagram of animal experiment. Bar plot showing (b) body weight, (c) the ratio of epididymal fat weight versus body weight, (d) the ratio of liver weight versus body weight, (e) ALT, (f) LDL, (g) TC, and (h) serum LPS levels of control and HFD-fed mice at weeks 4 and 12. (i) Protein levels of ZO-1 in colonic samples from control and HFD-fed mice at week 12 detected by IHC and western-blot analysis. (j) FITC-dextran intensity of control and HFD-fed mice after intervention for 12 weeks ⁣^∗^ and ⁣^∗∗^ means *p* < 0.05 and *p* < 0.01, respectively.

All procedures were performed in full compliance with the national standard of the Laboratory Animal Guideline for Ethical Review of Animal Welfare, the People's Republic of China National Standard. This research was specifically approved by the Animal Care and Use Committee of Hangzhou Normal University (Ethics Code Permit HSD20220801).  Figure 1(a) illustrates the schematic diagram of animal experiment. Concisely, 10 male C57BL/6J mice at the age of 6-8 weeks were obtained from the Animal Experiment Center of Hangzhou Normal University. They were housed in a specific pathogen-free (SPF) laboratory with free access to food and drinking water, and a 12 h dark cycle. The temperature was set 23 ± 1°C. The mice were randomly allocated into control (Ctrl, *n* = 5) and high-fat diet (HFD, *n* = 5) groups. In control group, the mice were fed a control diet (AIN-93G) from Dyets, which contains 15.8% kcal fat, 63.9% kcal carbohydrate, and 20.3% kcal protein. In HFD group, the mice were fed a high-fat diet (AMLN) containing 40% kcal fat, 40% kcal carbohydrate, and 20% kcal protein from Dyets. Detailed contents of AIN-93G and AMLN are shown in Tables S1 and S2, respectively. Blood samples were collected at weeks 4 and 12. At week 12, the feces, liver, colonic tissues, and mucosal samples from all mice were also harvested. All samples were stored at −80°C immediately until use. Serum LPS level was detected using commercial kits. The level of serum markers including alanine aminotransferase (ALT), aspartate aminotransferase (AST), low-density lipoprotein (LDL), total cholesterol (TC), and triglyceride (TG) was measured by 7180 Automatic Biochemical Analyzer (Hitachi, Tokyo, Japan).

### 2.2. Intestinal Permeability

The intestinal permeability of mice after 12 weeks of intervention was assessed by fluorescein isothiocyanate-dextran (FITC-dextran) (Sigma). Briefly, 0.6 mg/g FITC-dextran was administered to the mice intragastrically 30 min before anesthetized. After 30 min, the blood was collected by cardiac puncture, and fluorescent signals from isolated serum were examined by plate Multimode microplate Reader (Agilent BioTek, USA) at 488 nm.

### 2.3. Immunohistochemical (IHC) Assay

Immunohistochemistry assay was utilized to investigate the expression of zonula occludens-1 (ZO-1) protein in colonic tissues. The colonic tissues were embedded in paraffin and cut into 5-*μ*m-thick slices. The sections went through xylene transparency, ethanol dehydration, microwave high-pressure antigen repair, as well as goat serum blocking, and then incubated with ZO-1 antibody and the secondary antibody. After staining with 3,3′-diaminobenzidine (DAB) and hematoxylin, the images were acquired using a Leica microsystem.

### 2.4. Western Blot Analysis

The proteins were extracted from colon tissues by lysing in RIPA buffer and centrifugation. A commercial kit was used to determine the concentration of proteins. The samples were subjected to SDS-PAGE and then transferred to polyvinylidene fluoride (PVDF) membrane. The membranes were then incubated with polyclonal antibodies anti-ZO-1 and anti-GAPDH for overnight. After incubated with the secondary antibody for 1 h, the images were acquired with an automatic chemiluminescence analyzer.

### 2.5. DNA Extraction and Microbiome Sequencing

Bacterial total DNA was purified from brushed material of the colon and feces using the QIAamp DNA Micro kit (Qiagen) following manufacturer's instructions. The concentration and molecular size of bacterial DNA was assessed by NanoDrop (Thermo Scientific) and agarose gel electrophoresis, respectively. Workflows for DNA library construction such as cluster generation, template hybridization, isothermal amplification, denaturing and hybridization of sequencing primers was performed according to manufacturer's instructions (Illumina). Whole-genome metagenomic sequencing was performed at a read length of 250 base pairs (bp) in paired-end mode using Illumina HiSeq 4000 platform (Illumina).

### 2.6. Bioinformatic Analysis of Metagenomic Sequencing Data

Metagenomic sequencing data was preprocessed using the pipeline “sunbeam”. Firstly, adapter sequences were removed, and bases were quality filtered by Trimmomatic. Read pairs surviving quality filtering were then assessed for sequence complexity using Komplexity. Reads with sequence complexity higher than a default complexity threshold were then mapped against host sequences (mouse genome assembly GRCm39) using bwa. Reads that were mapped to any of these sequences within certain identity and length thresholds were removed. For either process, default parameters were used. After the above quality-control process, the decontaminated and quality-controlled reads were classified taxonomically using a Kraken2 database built on December 2022, and annotated functionally using Diamond and KEGG database. *α*-diversity indexes including Shannon and Simpson were calculated using the *R* package “vegan”. *β*-diversity between groups was estimated using principal coordinate analysis (PCoA) in *R* package “vegan” based on Bray–Curtis distance.

### 2.7. Co-Occurrence Network Analysis

We calculated the co-occurrence networks of mucosal microbiome for control and HFD-fed mice, respectively. With the profiles of mucosal microbiome for each group, we calculated the correlations between species using the Python module SparCC, and the correlations *r* > 0.4 or *r* < −0.4 (*p* < 0.05) were kept. The co-occurrence networks were then plotted using *R* package “igraph”. We also calculated the number of nodes and edges, degree distribution, average degree, and clustering coefficients in each network using “igraph”. In the co-occurrence network, each node indicated a species, and each edge indicated a relationship between species. Furthermore, the hub bacteria were defined when their degrees exceed the third quartile of the degree in a network.

### 2.8. Statistical Analysis

The continuous univariate data were presented as median and interquartile range (IQR). The differences between groups for univariate variables were assessed using Mann–Whitney *U* test. The overall mucosal microbiome composition between groups was compared using permutational multivariate analysis of variance (PERMANOVA). Microbiome and functional pathways that exhibited differential expression between groups were selected according to *p* values less than 0.05 from MaAslin2 [19] and LDA scores higher than 1.5 from LEfSe [20]. MaAslin2 estimated the multivariable associations between HFD and mucosal microbiome, while controlling the fixed effect of body weight. Associations between microbiome, serum markers and functional pathways were calculated using Spearman correlation coefficients based on “stats” package in *R*. Correlations with *p* values less than 0.05 were considered statistically significant. Unless otherwise specified, all statistical analyses were conducted using *R* software (version 4.3.3).

## 3. Results

### 3.1. HFD Induced Fat Accumulation and Intestinal Barrier Injury in Mice

We observed no significant increase in body weight of HFD-fed mice during HFD intervention at weeks 4 and 12 compared to corresponding controls (Figure 1(b)). Nevertheless, the ratio of epididymal fat weight to body weight elevated significantly from week 4 (Figure 1(c)). A significant increase in the ratio of liver weight to body weight was observed at week 12 (Figure 1(d)). Such results indicated that HFD intervention for 12 weeks induced fat accumulation in mice, although there was no significant weight gain.

As to serum markers, no significant perturbation was observed for either of them at week 4, while ALT, LDL, and TC levels elevated significantly at week 12 (Figures 1(e), 1(f), and 1(g)). We also observed that serum LPS level increased significantly in HFD-fed mice compared to corresponding controls at weeks 4 and 12 (Figure 1(h)), which implied the onset and persistence of intestinal barrier injury. As a further confirmation, we evaluated the intestinal permeability of HFD-fed mice after 12 weeks of intervention by assessing FITC-dextran intensity and ZO-1 protein expression in colonic tissues using both IHC assay and western-blot analysis. We observed significant decrease of ZO-1 protein in colonic tissues of HFD-fed mice based on both IHC assay and western-blot analysis (*p* < 0.01, Figure 1(i)). The FITC-dextran intensity, a measure of intestinal permeability, was also vividly elevated in HFD-fed mice (*p*=0.01, Figure 1(j)). In a word, HFD induced both fat accumulation and intestinal barrier injury in mice.

### 3.2. HFD Induced Mucosal Microbiome Reshape in Mice

**Figure 2:**
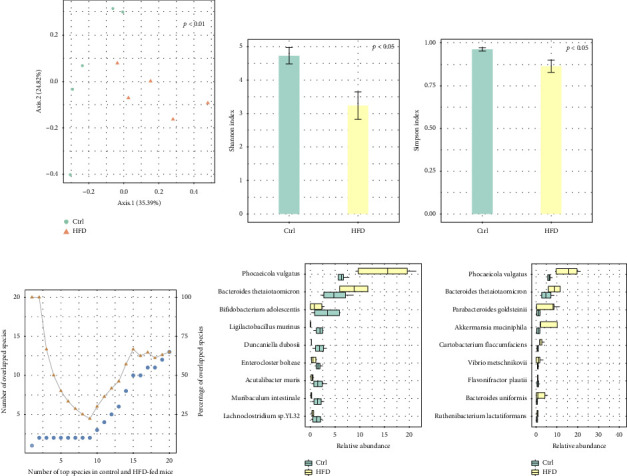
(a) Principal coordinate analysis of mucosal microbiome based on Bray–Curtis distances. Percentage of variance explained by each principal coordinate axis is shown in percentages. Bar plot of (b) Shannon index and (c) Simpson index in control and HFD-fed mice after intervention for 12 weeks. (d) The overlap of top 1 to top 20 species in control and HFD-fed mice in terms of relative abundance. (e) Boxplot of the 9 species that are most abundant in control mice. (f) Boxplot of the 9 species that are most abundant in HFD-fed mice.

We then explored the changes of mucosal microbiome in HFD-fed mice after 12 weeks of intervention based on colonic mucosal samples. PERMANOVA analysis detected a significant difference in mucosal microbiome composition between HFD-fed and control mice (*p* < 0.01) in terms of species. PCoA based on Bray–Curtis distance yielded consistent results. Specifically, the mucosal microbiome from HFD-fed and control mice clustered separately (Figure 2(a)). The Shannon and Simpson indexes (Figure 2(b) and 2(c)) demonstrated that the *α* diversity of mucosal microbiome in HFD-fed mice was significantly decreased compared to control mice. We then sorted the species according to their relative abundances in descending order, selected the top 1 to 20 species from HFD-fed and control mice separately, and evaluated their overlap. Among the top 9 species, only the top 2 species were shared by HFD-fed and control mice (Figure 2(d)).  Figure 2(e) illustrates the relative abundance of the 9 species that were most abundant in control mice. Among them, we found that the most abundant 2 species increased in HFD-fed mice, while the other 7 species decreased. The relative abundance of the 9 species that were most abundant in HFD-fed mice is shown in Figure 2(f). These findings suggested that HFD induced mucosal microbiome reshape in mice.

### 3.3. Detailed Reshape of Mucosal Microbiome in HFD-Fed Mice

**Figure 3:**
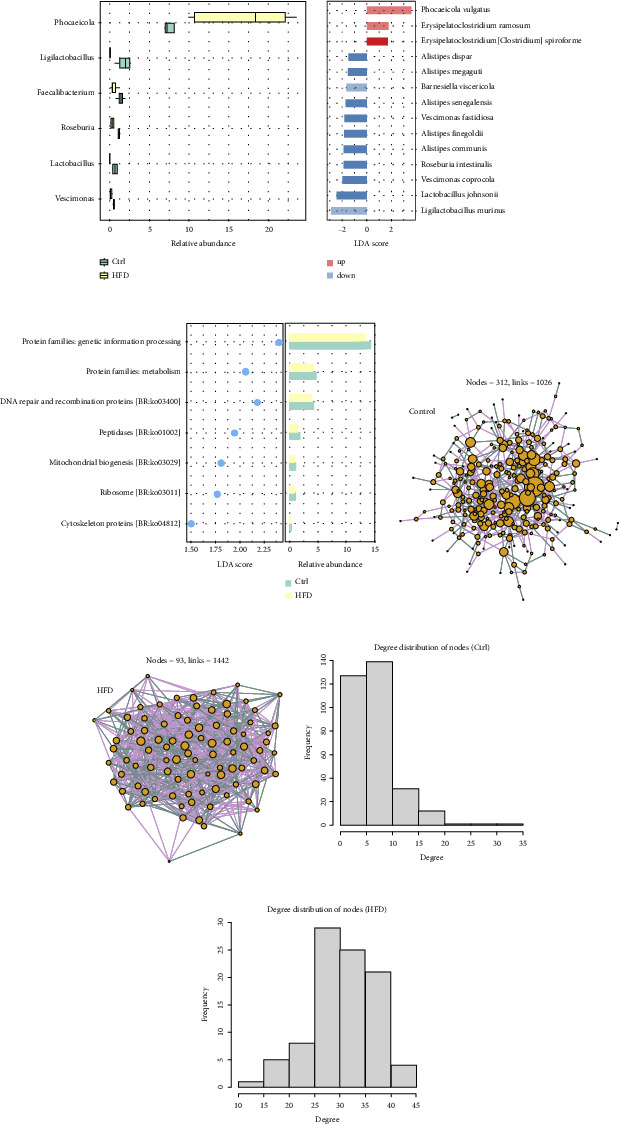
(a) Boxplot of the 6 genera differential between control and HFD-fed mice. (b) LDA scores of the 14 species differential between control and HFD-fed mice. (c) The LDA scores of the KEGG pathways differential between control and HFD-fed mice, as well as the relative abundances. (d) The co-occurrence network of mucosal microbiome in control mice. (e) The co-occurrence network of mucosal microbiome in HFD-fed mice. (f) Histogram showing the degree distribution of species in the co-occurrence network of control mice. (g) Histogram showing the degree distribution of species in the co-occurrence network of HFD-fed mice.

We then evaluated detailed reshape of mucosal microbiome in HFD-fed mice. Firstly, we examined the genera and species significantly modulated in HFD-fed mice compared to controls.  Figure 3(a) illustrates the relative abundance of the 6 genera differentially expressed between two groups, while Figure 3(b) shows the LDA scores for the 14 differential species. The LDA scores of differential genera, as well as the relative abundances of differential species, are shown in Figure S1. Concisely, genera *Phocaeicola* was significantly upregulated in HFD-fed mice, while the downregulated genera included *Ligilactobacillus*, *Faecalibacterium*, *Roseburia*, *Lactobacillus*, and *Vescimonas*. Species *Phocaeicola vulgatus*, *Erysipelatoclostridium ramosum*, and *Erysipelatoclostridium [Clostridium] spiroforme* were significantly elevated in HFD-fed mice, while 11 species such as *Alistipes dispar*, *Alistipes megaguti*, *Barnesiella viscericola*, *Vescimonas fastidiosa*, *Lactobacillus johnsonii*, and *Ligilactobacillus murinus* were significantly decreased. We also found that KEGG pathways such as “Protein families: metabolism”, “DNA repair and recombinant proteins”, and “Mitochondrial biogenesis” were significantly downregulated in HFD-fed mice compared to controls (Figure 3(c)).

Considering that microbial communities involve complex synergistic and antagonistic relationships [21], we constructed mucosal microbiome co-occurrence networks for control (Figure 3(d)) and HFD-fed mice (Figure 3(e)) separately. We also applied the complex network approach for the topological properties and hub bacteria of the networks. The microbial species and their correlations were considered as nodes and links in the mucosal microbiome networks. The degree of a node meant the number of edges connected to the node. Based on the degree distribution of nodes shown in Figure 3(f), we discovered that the distribution histogram of the network in control mice tended to fit the power-law model, and demonstrated scale-free features. The degree distribution of the nodes in HFD-fed mice showed characteristics of Poisson model (Figure 3(g)). Such results indicated that the mucosal microbiome networks changed from scale-free networks in control mice to nearly random graphs in HFD-fed mice. In addition, the control mice had more nodes compared to HFD-fed mice (Figures 3(d) and 3(e)). It is likely related to the higher bacterial diversity in control mice. Nevertheless, the microbial network in HFD-fed mice demonstrated more links, higher average degree, and clustering coefficients (Table S3). Moreover, it was revealed that the network in control mice was accompanied by more hub bacteria (*n* = 73) than the network in HFD-fed mice (*n* = 13). In a word, HFD induced reshape in composition of species and functions, as well as mucosal microbiome network.

### 3.4. Associations Between Mucosal Microbiome Reshape and HFD-Related Intestinal Barrier Injury

**Figure 4:**
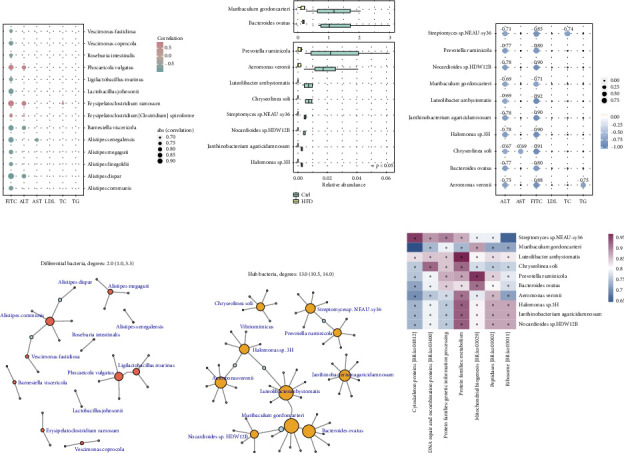
(a) Bubble plot showing the association between 14 differential species and serum markers. (b) Boxplot illustrating the relative abundance of 10 hub species in control mice. (c) Heatmap illustrating the associations between 10 hub species in control mice and serum markers. Networks associated with (d) 14 differential bacteria and (e) 10 hub bacteria. The red and orange nodes represent differential and hub bacteria separately, while blue nodes are bacteria regulated by them. (f) Heatmap of the associations between 10 hub species in control mice and differential KEGG pathways.

The associations between mucosal microbiome reshape and HFD-related intestinal barrier injury were evaluated based on both differential and hub bacteria. Firstly, we probed the correlations between 14 differential species and FITC-dextran intensity. It was found that all the 14 species correlated significantly with FITC-dextran intensity (Figure 4(a)). Among them, the three species upregulated in HFD-fed mice, including *Phocaeicola vulgatus*, *Erysipelatoclostridium ramosum*, and *Erysipelatoclostridium [Clostridium] spiroforme*, showed positive correlations with FITC-dextran intensity, while the other 11 species downregulated in HFD-fed mice, such as *Roseburia intestinalis, Vescimonas fastidiosa*, and *Lactobacillus johnsonii*, correlated negatively with FITC-dextran intensity. Additionally, the 14 differential bacteria also showed significant correlations with ALT (Figure 4(a)). Then, we explored hub bacteria in mucosal microbiome co-occurrence networks. We found that 10 hub bacteria in control mice including *Streptomyces* sp. *NEAU-sy36*, *Luteolibacter ambystomatis*, *Janthinobacterium agaricidamnosum*, *Nocardioides* sp. *HDW12B*, *Halomonas* sp. *3H*, *Aeromonas veronii*, *Bacteroides ovatus*, *Prevotella ruminicola*, *Chryseolinea soli*, and *Muribaculum gordoncarteri* were all downregulated in mucosal samples of HFD-fed mice (Figure 4(b)). Majority of them were also decreased in fecal samples of HFD-fed mice (Figure S2a). They also showed significant negative correlations with FITC-dextran intensity and ALT (Figure 4(c)). Such results indicated that both differential and hub bacteria were associated with intestinal permeability and maybe organ dysfunction.

We further evaluated the capability of differential and hub bacteria in regulating microbial community. Figures 4(d) and 4(e) illustrate the microbiota regulated by differential and hub bacteria, respectively. The red nodes (Figure 4(d)) and orange nodes (Figure 4(e)) represented differential and hub bacteria separately, while the blue nodes were bacteria regulated by them. Moreover, the size of a node was proportional to its degree. It was observed that the average degree of hub bacteria was significantly higher than those of differential bacteria (Figure S2b). Such results indicated that hub bacteria can be much more efficient in regulating microbial community, when utilized as probiotics. We also observed significant positive correlations between hub bacteria and pathways such as “Protein families: metabolism” (Figure 4(e)). Thus, it can be proposed that the decrease of hub bacteria contributed to impaired metabolic capacity of microbial system, which will then lead to the lost control of them in maintaining the homeostasis of mucosal microbial community. In a word, mucosal hub bacteria may provide targets with better robustness and globality in terms of microbial community regulation for HFD-related intestinal barrier injury.

## 4. Discussion

To unravel the associations between mucosal hub bacteria and intestinal barrier injury, we induced intestinal barrier injury in mice with HFD, and characterized the reshape of mucosal microbiome using whole-genome metagenomics. Our investigation revealed substantial alterations in both the composition and co-occurrence networks of mucosal microbiome in HFD-fed mice. Beyond the species differentially expressed between HFD-fed and control mice, some mucosal hub bacteria prevalent in controls were drastically decreased in both mucosal and fecal samples of HFD-fed mice, and were found to be associated with intestinal barrier injury.

Currently, candidate probiotics for new therapeutics are defined as those which differ in abundance between healthy and diseased ecosystems. Yet, such microbial therapeutics may end in inconsistent performance, since not all transplanted species can establish in every recipient [22], and dysbiotic microbiome are often more variable than healthy ones [12]. Thus, we tried microbial networks, which are superior in representing emergent properties that can only arise from the collaborative functioning of a system [23]. We found in this study that 10 hub bacteria in control mice such as *Luteolibacter ambystomatis*, *Aeromonas veronii*, and *Bacteroides ovatus* decreased in mucosal samples of HFD-fed mice. It was reported that *Bacteroides ovatus* can reestablish the equilibrium between lymphocytes and macrophages, and maintain the balance of immune system [24]. Outer membrane vesicles secreted by *Bacteroides ovatus* was found to carry inulin-degrading enzymes, which can produce nutrients for *Bacteroides* species that cannot utilize inulin [25]. *Bacteroides ovatus* was also found to reduce the degradation of colonic mucus by other commensals such as *Bacteroides thetaiotaomicron* by metabolizing dietary polysaccharides into monosaccharides [26]. Thus, the absence of *Bacteroides ovatus* may lead to the dysbiosis of intestinal microecology, death of intestinal epithelial cells and inflammation [24]. *Prevotella ruminicola* is one of the cellulolytic bacteria. It can participate in the degradation of hemicellulose into mainly succinate, acetate, and propionate through hydrolysis of xylan [27, 28]. Studies indicated that addition of several probiotic bacteria including *Prevotella ruminicola* to the diet can restore gut microbiota balance, and lower oxidative stress in gut and brain [29]. Although recognized as pathogens, some bacteria are also key members in maintaining the balanced gut and mucosal microbial system. For example, *Aeromonas veronii*, a common member of zebrafish intestinal microbiota [30], secreted N-acetylglucosamine (GlcNAc) binding protein N-acetylglucosamine-binding protein A (GbpA), that was sufficient to stimulate epithelial cell proliferation in the zebrafish intestine [31]. Such results indicated that hub bacteria played key roles in maintaining the homeostasis of microbial community. Combined with the significant correlations between hub bacteria and intestinal permeability, it is reasonable to propose that mucosal hub bacteria associate with HFD-related intestinal barrier injury.

Furthermore, the above 10 hub bacteria were drastically decreased in both mucosal and fecal samples of HFD-fed mice, and positive correlations were observed between them and KEGG pathways such as “metabolism”. Such results implied that the decrease of the above hub bacteria may contribute to impaired metabolic capability of the microbial community, which will then lead to the lost control of hub bacteria in maintaining the homeostasis of microbial community, and finally intestinal barrier injury. Moreover, the superiority of hub bacteria over differential bacteria as targets was revealed by higher degrees in networks, indicators of capability in regulating microbial community. Nowadays, the value of hub nodes in networks has been confirmed by successful identification of multiple new drug targets by Emig et al. [32] and new functional modules in plant science by Taylor-Teeples et al. [33] Most importantly, *Bacteroides ovatus*, the most abundant hub bacteria identified in this study, has been confirmed to improve intestinal barrier function in HFD-fed mice by activating aryl hydrocarbon receptor with the key bioactive metabolite indoleacetic acid [34]. Therefore, hub bacteria that lost control in HFD-fed mice may act as potential targets for improving HFD-related intestinal barrier injury.

However, it should be noted that the efficacy of probiotics is quite limited in patients with severe intestinal microbiome disturbance [35, 36], probably due to the drastic difference between the microbial networks in healthy and diseased states. Compared to reconstructing healthy microbial networks from those dysregulated ones, inhibiting or reversing the shifting process in early stages of diseases based on microbial manipulation may be much easier. Thus, attentions should be paid to milder cases of diseases.

## 5. Conclusions

Our study revealed that both differential and hub bacteria in mucosal microbiome were closely linked to HFD-related intestinal barrier injury, while hub bacteria with higher degrees in microbial networks showed apparent superiority in microbial community regulation. These hub bacteria engage with other commensals in safeguarding mucosal barrier function, and may play a pivotal role in restoring microbial equilibrium. Consequently, hub bacteria within the microbial network could serve as promising targets for enhancing the intestinal barrier function associated with HFD. However, the potential of probiotics to reconstruct a healthy microbial network from severely dysregulated ones may be limited. Further research studies evaluating the efficacy of microbial manipulation in improving intestinal barrier injury, especially those targeting on hub bacteria, should focus on milder cases.

## Data Availability

The data that support the findings of this study are openly available in Sequence Read Archive at https://www.ncbi.nlm.nih.gov/sra, reference number PRJNA1034789.

## Nomenclature

ALT: Alanine transaminase
AST: Aspartate aminotransferase
FITC: Fluorescein 5-isothiocyanate
HFD: High-fat diet
LDL: Low-density lipoprotein
NAFLD: Nonalcoholic fatty liver disease
NASH: Nonalcoholic steatohepatitis
TC: Total cholesterol
TG: Triglyceride
ZO-1: Zonula occludens-1

## Supporting Information

Supporting Information Table S1. Dietary ingredient composition of AIN-93G diet.

Supporting Information Table S2. Dietary ingredient composition of AMLN diet.

Supporting Information Table S3. Characteristics of co-occurrence networks in HFD-fed and control mice.

Supporting Information Figure S1. (a) LDA scores for differential genera. (b) Relative abundances of differential species upregulate in HFD-fed mice. (c) Relative abundances of differential species downregulated in HFD-fed mice.

Supporting Information Figure S2. (a) The relative abundance of the 10 mucosal hub species in fecal samples. (b) Bar plot showing the degree of differential and hub bacteria.
