# Supplementary material for: Mucosal Hub Bacteria as Potential Targets for Improving High-Fat Diet-Related Intestinal Barrier Injury
Source: Can J Infect Dis Med Microbiol. 2024 Nov 27;2024:3652740. doi: 10.1155/cjid/3652740 (PMC11617042; doi:10.1155/cjid/3652740)
Supplement: Supporting Information — Table S1. Dietary ingredient composition of AIN-93G diet. [file 3652740.f1.docx]

**Table S1**. Dietary ingredient composition of AIN-93G diet.

| Ingredient | weight (g/kg) |
| --- | --- |
| Corn sugar | 397.5 |
| Casein lactic | 200 |
| Granular sugar | 100 |
| Dextrin | 132 |
| Solka floc-40 | 50 |
| AIN-93 mineral mix | 35 |
| AIN-93 vitamin mix | 10 |
| L-cystine | 3 |
| Choline bitartrate | 2.5 |
| Soy oil | 70 |
