# Supplementary material for: Mucosal Hub Bacteria as Potential Targets for Improving High-Fat Diet-Related Intestinal Barrier Injury
Source: Can J Infect Dis Med Microbiol. 2024 Nov 27;2024:3652740. doi: 10.1155/cjid/3652740 (PMC11617042; doi:10.1155/cjid/3652740)
Supplement: Supporting Information — Table S2. Dietary ingredient composition of AMLN diet. [file 3652740.f2.docx]

**Table S2.** Dietary ingredient composition of AMLN diet.

| Ingredient | gm |
| --- | --- |
| Casein | 200 |
| L-cystine | 3 |
| Maltodextrin 10 | 100 |
| Fructose | 200 |
| Sucrose | 96 |
| Cellulose | 50 |
| Soybean oil | 25 |
| Lard | 20 |
| Palm oil | 135 |
| Mineral mix S10026 | 10 |
| DiCalcium phosphate | 13 |
| Calcium carbonate | 5.5 |
| Potassium citrate, 1 H2O | 16.5 |
| Vitamin mix V10001 | 10 |
| Choline bitartrate | 2 |
| Cholesterol | 18 |
| FD&C Red Dye #40 | 0.025 |
| FD&C Blue Dye #1 | 0.025 |
