# Supplementary material for: Mucosal Hub Bacteria as Potential Targets for Improving High-Fat Diet-Related Intestinal Barrier Injury
Source: Can J Infect Dis Med Microbiol. 2024 Nov 27;2024:3652740. doi: 10.1155/cjid/3652740 (PMC11617042; doi:10.1155/cjid/3652740)
Supplement: Supporting Information — Table S3. Characteristics of co-occurrence networks in HFD-fed and control mice. [file 3652740.f3.docx]

**Table S3**. Characteristics of co-occurrence networks in HFD-fed and control mice.

|  | Number of nodes | Number of edges | average degree | clustering coefficient | Number of hub bacteria |
| --- | --- | --- | --- | --- | --- |
| control | 312 | 1026 | 6.577 | 0.074 | 73 |
| HFD | 93 | 1442 | 31.011 | 0.204 | 13 |
