# Supplementary figures and images for: Mucosal Hub Bacteria as Potential Targets for Improving High-Fat Diet-Related Intestinal Barrier Injury
Source: Can J Infect Dis Med Microbiol. 2024 Nov 27;2024:3652740. doi: 10.1155/cjid/3652740 (PMC11617042; doi:10.1155/cjid/3652740)

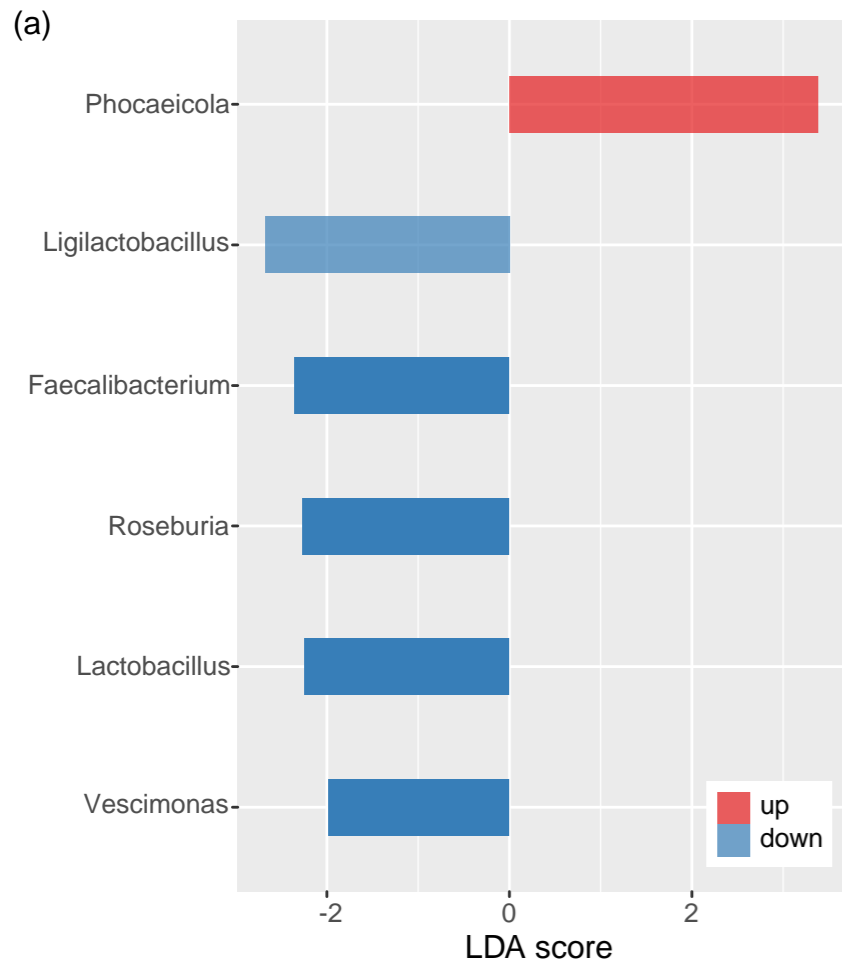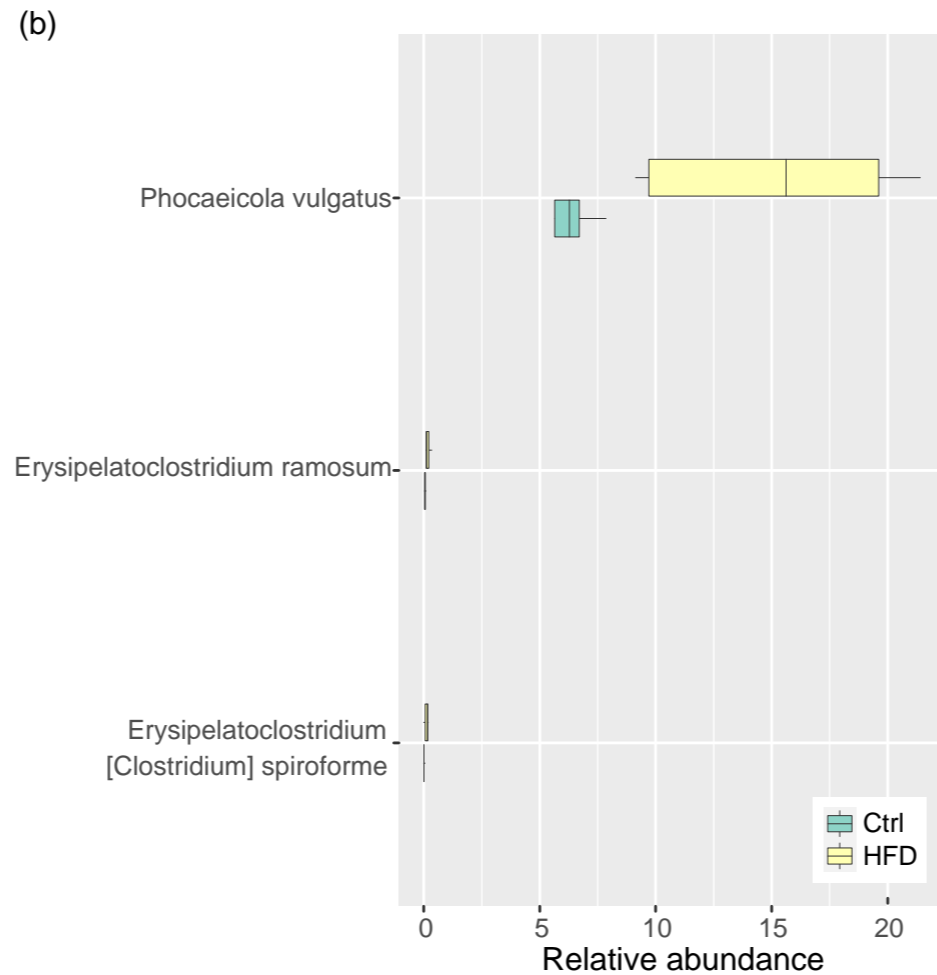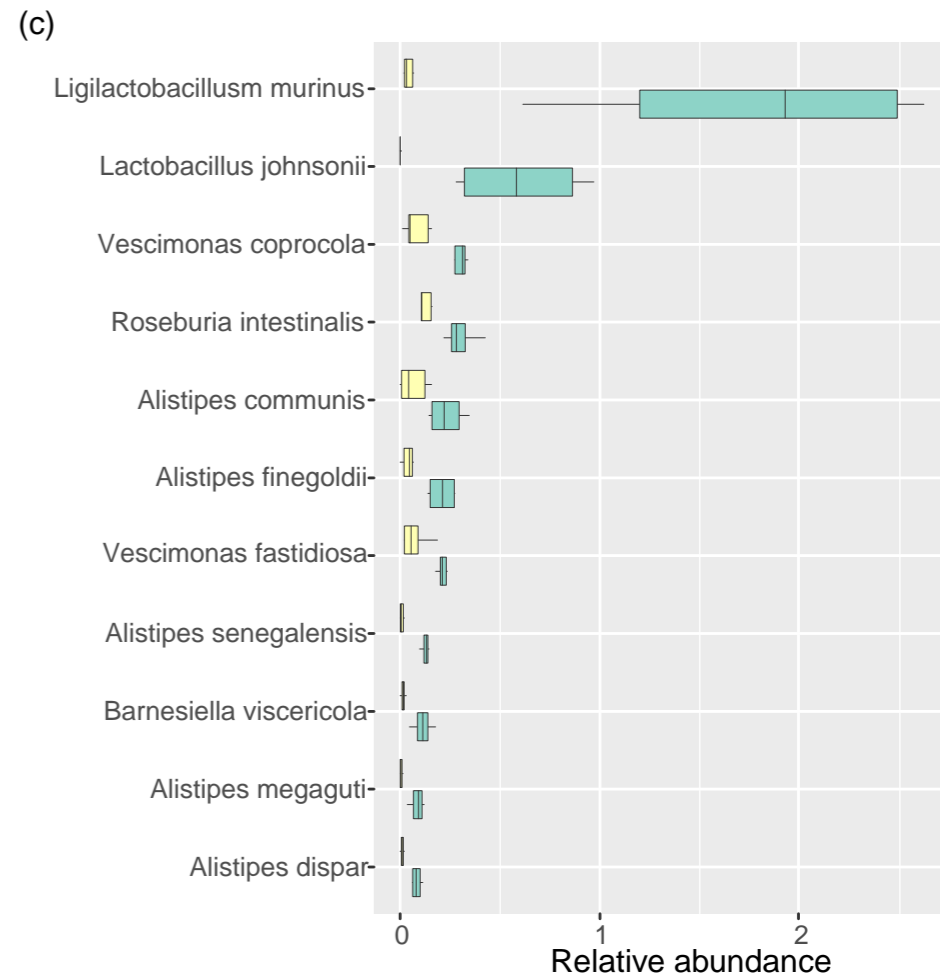

Supplement: Supporting Information — Figure S1. (a) LDA scores for differential genera. (b) Relative abundances of differential species upregulate in HFD-fed mice. (c) Relative abundances of differential species downregulated in HFD-fed mice. [file 3652740.f4.pdf]

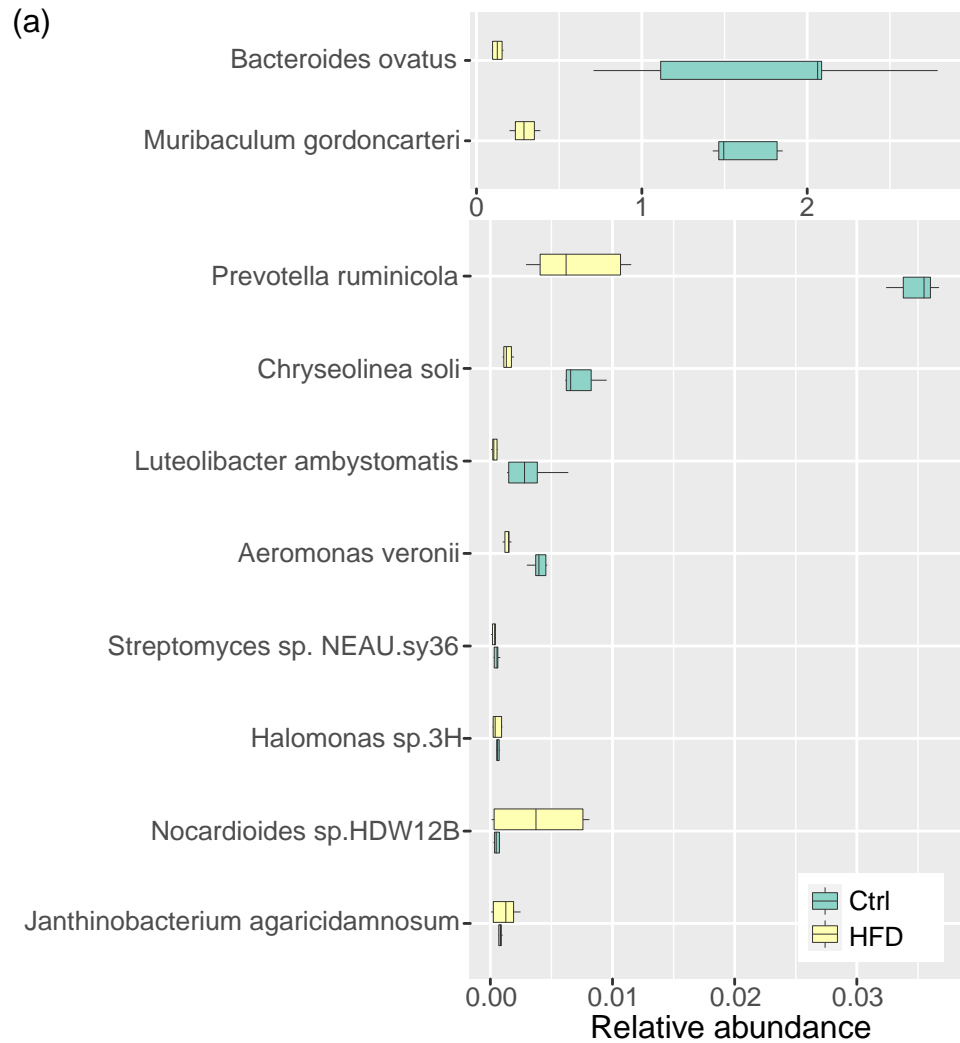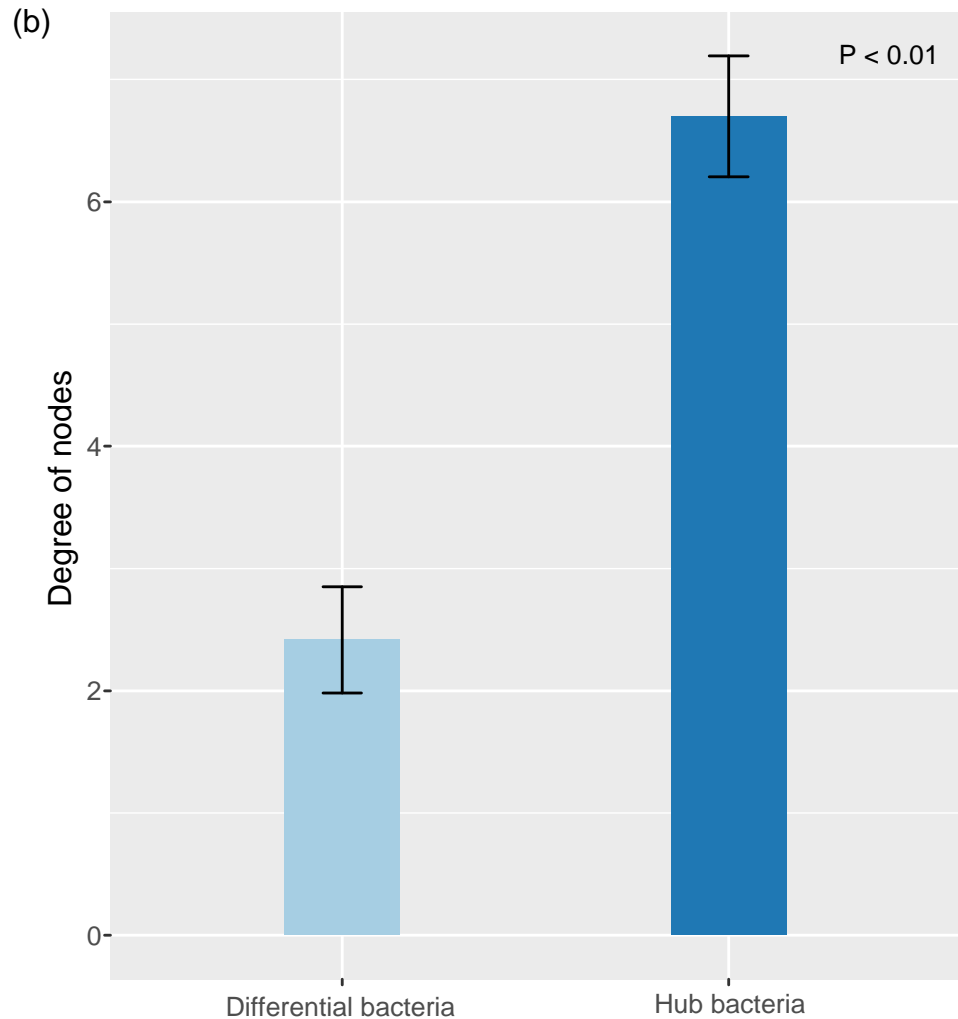

Supplement: Supporting Information — Figure S2. (a) The relative abundance of the 10 mucosal hub species in fecal samples. (b) Bar plot showing the degree of differential and hub bacteria. [file 3652740.f5.pdf]
